# Effectiveness of exercise in office workers with neck pain: A systematic review and meta-analysis

**DOI:** 10.4102/sajp.v73i1.392

**Published:** 2017-11-28

**Authors:** Shereen Louw, Shale Makwela, Lorisha Manas, Lyle Meyer, Daniele Terblanche, Yolandi Brink

**Affiliations:** 1 Department of Health and Rehabilitation Sciences, Stellenbosch University, South Africa

## Abstract

**Background:**

Non-specific neck pain is a common health problem of global concern for office workers. This systematic review ascertained the latest evidence for the effectiveness of therapeutic exercise versus no therapeutic exercise on reducing neck pain and improving quality of life (QoL) in office workers with non-specific neck pain.

**Method:**

Seven electronic databases using keywords, that is, ‘office workers’, ‘non-specific neck pain’, ‘exercise’ and/or ‘exercise therapy’, ‘QoL’, ‘strengthening’, ‘stretching’, ‘endurance’, ‘physiotherapy’ and/or ‘physical therapy’, were searched from inception until March 2017. Heterogeneous data were reported in narrative format and comparable homogenous data were pooled using Revman.

**Results:**

Eight randomised control trials were reviewed and scored on average 6.63/10 on the Physiotherapy Evidence Database (PEDro) scale. Five studies performed strengthening exercise, one study had a strengthening and an endurance exercise group, one study performed stretching exercise and one study had an endurance intervention group and a stretching intervention group. Five and four studies reported significant improvement in neck pain and QoL, respectively, when conducting strengthening exercise. When performing endurance exercises, one and two studies reported significant changes in neck pain and QoL, respectively. The one study incorporating stretching exercise reported significant improvement in neck pain. The meta-analysis revealed that there is a clinically significant difference favouring strengthening exercise over no exercise in pain reduction but not for QoL.

**Conclusion:**

There is level II evidence recommending that clinicians include strengthening exercise to improve neck pain and QoL. However, the effect of endurance and stretching exercise needs to be explored further.

## Introduction

Non-specific neck pain is a common health problem of global concern especially in office workers (Hanvold et al. 2014). Neck pain is classified as non-specific when the pathophysiology is relatively unknown or unclear. The pain is not because of any underlying pathology or systemic disease; however, symptoms are localised to the neck region (Sihawong et al. 2011; Verhagen et al. 2013). Work-related musculoskeletal pain has a high prevalence rate of self-reported non-specific neck pain in both developed and developing countries (Cagnie et al. 2007). In Australia, a one-year incidence rate of 49% was recorded for neck pain in office workers (Hush et al. 2009). In developing countries, such as India, Shah and Patel (2015) reported a 47% prevalence rate for office-related neck pain. In South Africa, there is limited published research on the prevalence of work-related neck pain; however, Zungu and Ndaba (2009) reported that 28.2% of office workers complained of pain localised mostly to the neck, shoulder and wrist areas. The above evidence indicates that non-specific neck pain in office workers could be of concern over multiple continents.

Neck pain has been associated with decreased health-related quality of life (HRQoL) in several studies (Cook & Harman 2008; Lobbezoo, Visscher & Naeije 2004; Luo et al. 2004; Saarni et al. 2006). In 2007, office workers in the Netherlands presented with a 31% decrease in their quality of life (QoL) scores after they started experiencing neck pain (Sluiter & Frings-Dresen 2007). Non-specific neck pain is often the cause of working days lost (Verhagen et al. 2013). Work-related musculoskeletal disorders (WRMSDs) accounted for 41% of the prevalence of all work-related ill health in Great Britain in 2015/2016; WRMSDs working days lost (which place burdens on employers) accounted for 34% of all days lost because of work-related illness; work-related upper limb disorders (WRULDs) because of keyboard or repetitive action accounted for 56 000 cases (27%) and awkward or tiring positions for 29 000 cases (14%) (Buckley 2016). Many measurement tools have been used, such as the Short Form-36 Health Survey (SF-36) or subscales of the SF-36, Neck Disability Index (NDI), 15-Dimensional HRQoL instrument (15D) and amount of Healthy or Sick Leave Days to assess QoL among patients with neck pain, but no gold standard measurement exists (Salo et al. 2010).

The most common reason for work absenteeism among office workers is because of pain or tenderness in the neck region, inhibiting working abilities (Hoe et al. 2012). Environmental (prolonged static or awkward postures, highly repetitive movements and computer work) and physical (inadequate strength or muscle endurance and poor posture) factors contribute to the development of work-related neck pain (Hoe et al. 2012; Verhagen et al. 2013). Computer workers are two to three times more likely to develop chronic neck pain when compared to the general population (Green 2008). Zungu and Ndaba (2009) reported that South African office workers, who spend 75% or more of the working day on a computer, have an increased risk of developing neck pain. Three studies found an association between computer work, poor posture (forward head postural alignment), cervical flexor and extensor muscle imbalances, muscle fatigue and the development or worsening of work-related neck pain (Falla, Jull & Hodges 2004; Hush, Refshauge & Maher 2006; Owen et al. 2010). Therefore, addressing these contributing factors to work-related neck pain will help reduce the onset and intensity of work-related neck pain and the absenteeism of office workers (Gerr et al. 2005).

Various treatment strategies have been implemented to successfully manage individuals with work-related neck pain to allow them to return to work as soon as possible (State Insurance Regulatory Authority 2017). Verhagen et al. (2013) showed that physiotherapy interventions such as exercise, mobilisation and electrotherapy modalities reduce work-related neck pain levels and improve function. This is achieved by re-educating, strengthening and stretching muscles; mobilising soft tissue and improving ergonomics and kinetic handling in the workplace. Sihawong et al. (2011) indicated that both strengthening and endurance exercises have superior benefits over stretching programmes for treatment of non-specific neck pain in office workers. O’Riordan et al. (2014) reported that resistance exercises and endurance training reduced pain and disability scores in office workers with chronic neck pain. Previous studies also revealed that office workers who received non-specific exercise, compared to receiving an educational pamphlet on ergonomics in the workplace, experienced a significant reduction in intensity and the duration of neck pain (Blangsted et al. 2008; Hanney et al. 2010).

Since the systematic review by Sihawong et al. (2011), more recent randomised controlled trials (RCTs) have been published, which focus on the effectiveness of exercise therapy in decreasing pain levels in office workers with non-specific neck pain. It is of importance to re-evaluate the current evidence for the effectiveness of exercise therapy in improving pain intensity and broadening the scope of effectiveness by including QoL as an outcome. QoL indicates the ability to perform activities of daily living (ADLs) and measures an individual’s independence (Kim, Kim & Kim 2014). Currently there is a lack of systematic reviews which measure QoL of office workers with non-specific neck pain. Therefore, this systematic review aimed to determine whether exercise therapy focusing on curative techniques (such as strength, endurance or stretching exercise) is effective to reduce pain and improve QoL in office workers with non-specific neck pain.

## Methodology

This systematic review was conducted according to the Preferred Reporting Items for Systematic Reviews and Meta-Analyses (PRISMA) guidelines (Moher et al. 2009).

### Eligibility criteria

Randomised controlled trials published in English and presented in full text were eligible for inclusion in the systematic review. Studies that included male and female populations, 18 years and older, participants actively working in offices and who had been diagnosed with or self-reported acute or chronic non-specific neck pain were included in the systematic review. The interventions provided in the studies consisted of exercise therapy, which included, but was not limited to, strengthening, stretching or endurance exercises. The comparison treatment provided to participants included, but was not limited to, no exercise or health promotional activities only. The health promotional activities could include education on ergonomics, stress management strategies or making general healthy lifestyle choices. Appropriate outcome measures for pain and QoL could include, but were not limited to, measures such as the Numerical Pain Rating Scale (NPRS) and the Visual Analogue Scale (VAS) to evaluate pain, and the NDI, the Short Form Questionnaire (SF-36) and the Disability of Arm, Shoulder and Hand Questionnaire (DASH) to evaluate QoL. Studies that were not classified as RCT, such as studies with an observational or descriptive design, were excluded from the review. Participants who were younger than 18 years; who were not working in an office set-up and diagnosed with neck pain not classified as non-specific neck pain but pain because of trauma, abnormalities in bones and joints, degenerative diseases or tumours; and who had a history of severe trauma or the presence of inflammatory rheumatic disorders were excluded. Interventions which provided manual therapy techniques or medication were not eligible for inclusion. Measurement tools that did not determine pain or QoL such as strength or range of motion were also not considered in this review.

### Information sources

All databases were accessed through the Stellenbosch University Library service. The databases included Physiotherapy Evidence Database (PEDro), PubMed, CINAHL, Science Direct, Cochrane Library, Scopus and Biomed Central. Preliminary searches were performed to exclude search terms that did not yield meaningful results. Search terms included ‘office workers’, ‘neck pain’, ‘exercise’ and/or ‘exercise therapy’, ‘quality of life’, ‘strengthening’, ‘stretching’, ‘endurance’, ‘physiotherapy’ and/or ‘physical therapy’. Each database’s advanced search function was designed according to their search strategy function.

### Search strategy

The seven databases were shared among the research team and each member was assigned to more than one database. All databases were searched from inception to 28 March 2017. Titles were either included or excluded based on the criteria for study eligibility. Authors searching the same database conferred their results on all included and excluded titles. If consensus was not reached, it was debated with other members of the research team. The same procedure for including titles was applied to selected abstracts and full text articles. All research team members read the included articles to confirm eligibility of those selected. Secondary searching was performed by viewing the included papers’ reference lists (PEARLing) for additional relevant sources.

### Methodological appraisal

The PEDro scale was used to critically appraise the methodological quality of the respective studies (Verhagen et al. 1998). The PEDro scale contains 11 items of which only 10 items are scored (YES/NO) (PEDro 1999). This scale appraises the level of quality of randomised control trials. The scale assesses external validity (criterion 1), internal validity (criteria 2–8) and statistical accuracy (criteria 9–10). It is scored as either present (1) or absent (0). However, the PEDro scale does not measure the clinical significance or the treatment effectiveness of a study. Three authors independently scored each included article. In the case of discrepancy between scores, the discrepancies were firstly discussed within the group, and if consensus could not be reached, then the article was reassessed according to the PEDro administration points criterion to reach a consensus (PEDro, 1999).

### Data extraction and analysis

Data from the selected articles were extracted using the adapted Joanna Briggs Institute (JBI) data extraction sheets (Pearson, Field & Jordan 2009). The following categories were extracted: article reference, study method, participants, intervention type, control type, clinical outcome measures, results and the clinical consequences (Pearson et al. 2009). Two authors were assigned to each article and independently extracted the appropriate data. They compared their findings to verify whether all appropriate data were successfully extracted. Authors of the articles were contacted for missing information. Two of the eight studies’ authors provided results obtained during their study to include in this review that were not reported in the article they published (Salo et al. 2010; Tunwattanapong, Kongkasuwan & Kuptniratsaikul 2015). The results from the studies where the data could not be summarised via meta-analysis techniques because of the varied duration of the interventions and outcome measurement scales used are presented in narrative (text and tables) format. Statistical inferences (*p*-values) of these studies were assessed to determine the effectiveness of the intervention. Results from the studies with comparable data, in terms of the methodology used, were combined using the Revman Review Manager software and are graphically presented using forest plots (Revman 2012).

## Results

### Study selection

**FIGURE 1:**
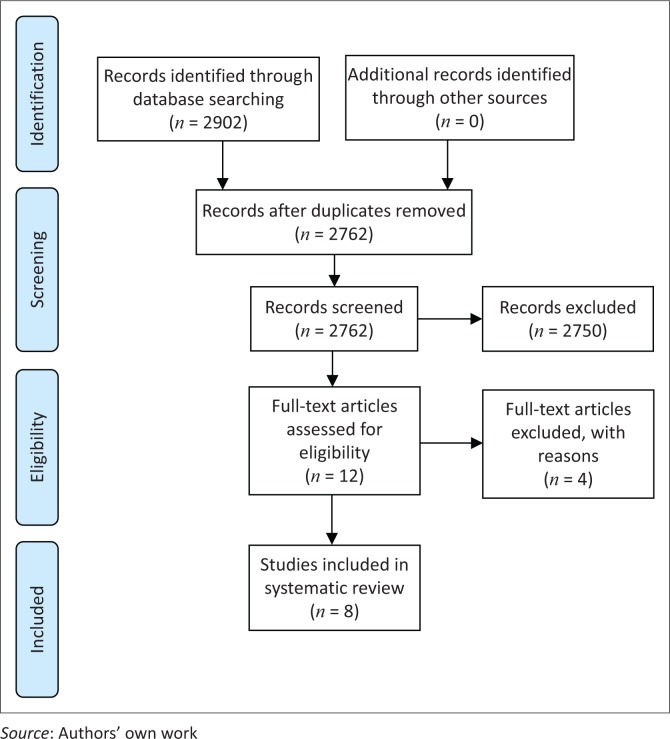
Preferred reporting items for systematic reviews and meta-analyses flow diagram showing the search results and inclusion of the studies.

A total number of 2902 initial hits were found. Figure 1 shows the initial hits and excluded duplicates (*n* = 140), titles (*n* = 2564), abstracts (*n* = 186) and full text articles (*n* = 4). Titles matching our search strategy were selected for potential inclusion and amounted to 198 (*n* = 198). Within each database, these abstracts were screened for eligibility and 186 abstracts were excluded. Thus, 12 potentially eligible full text articles were included. These full text articles were assessed according to the inclusion and exclusion criteria. Four full text articles were not eligible and thus excluded (two studies measured body pain and not only neck pain; one study did not report on pain and one study did not limit the sample to office workers only). Therefore, eight full text articles were eligible for this systematic review. The search strategy results are illustrated in Figure 1.

### Methodological appraisal

The methodological quality of the eight included articles was assessed using the PEDro scale. The total score ranged between 5 and 8 out of 10 with an average score of 6.63. Table 1 provides the results of the PEDro scores.

**TABLE 1:** Scores according to Physiotherapy Evidence Database criteria.

Author	Item 1	Item 2	Item 3	Item 4	Item 5	Item 6	Item 7	Item 8	Item 9	Item 10	Item 11	Total Score
Andersen et al. (2008)	Y	Y	N	N	N	N	N	Y	Y	Y	Y	6/10
Andersen et al. (2011)	Y	Y	Y	Y	N	N	Y	Y	Y	Y	Y	8/10
Andersen et al. (2012)	Y	Y	N	Y	N	N	N	N	Y	Y	Y	5/10
Gram et al. (2014)	Y	Y	N	Y	N	N	N	N	Y	Y	Y	5/10
Nikander et al. (2006)	Y	Y	N	Y	N	N	Y	Y	N	Y	Y	6/10
Salo et al. (2010)	Y	Y	Y	Y	N	N	Y	Y	N	Y	Y	7/10
Tunwattanapong et al. (2015)	Y	Y	Y	Y	N	N	Y	Y	Y	Y	Y	8/10
Viljanen et al. (2003)	Y	Y	Y	Y	N	N	Y	Y	Y	Y	Y	8/10

*Source*: Authors’ own work

1. Eligibility criteria were specified (not included in the score).

2. Participants were randomly allocated to groups (in a crossover study, subjects were randomly allocated in order in which treatments were received).

3. Allocation was concealed.

4. The groups were similar at baseline regarding the most important prognostic indicators.

5. There was a blinding of all participants.

6. There was a blinding of all therapists who administered the therapy.

7. There was blinding of all assessors who measured at least one key outcome.

8. Measures of at least one key outcome were obtained from more than 85% of the participants initially allocated to groups.

9. All participants for outcome measures were available received the treatment or control condition as allocated or, where this was not the case, data for at least one key outcome were analysed by ‘intention to treat’.

10. The results of between-group statistical comparisons are reported for at least one key outcome.

11. The study provides both point measures and measures of variability for at least one key outcome.

### Study sample description

The sample descriptions in terms of sample size and gender are reported in Table 2. Three of the eight studies only included female participants: Viljanen et al. (2003), Nikander et al. (2006) and Salo et al. (2010). The sample size of the eight studies ranged from 96 to 449 participants and the mean ages from 34.2 to 49 years old.

**TABLE 2:** Study sample description.

Author	Sample size				Gender				Age Mean (SD)				Significant differences reported at baseline
	Contr	Int 1	Int 2	Int 3	Contr	Int 1	Int 2	Int 3	Contr	Int 1	Int 2	Int 3	
Andersen et al. (2008)	182	180	-	-	M: 42 F: 138	M: 41 F: 139	-	-	45.5 (N/R)	46.5 (N/R)	-	-	N/R
Andersen et al. (2011)	64	65	63	-	M: 8 F: 58	M: 8 F: 58	M: 8 F: 58	-	43 (10.0)	42 (11.0)	44 (11.0)	-	N/R
Andersen et al. (2012)	101	116	126	106	M: 42 F: 59	M: 44 F: 72	M: 39 F: 87	M: 45 F: 61	46 (10.0)	47 (10.0)	46 (10.0)	45 (10.0)	N/R
Gram et al. (2014)	101	126	124	-	M: 42 F: 59	M: 39 F: 87	M: 52 F: 72	-	46 (10.0)	46 (10.0)	45 (11.0)	-	No significant difference
Nikander et al. (2006)	60	60	60	-	M: 0 F: 60	M: 0 F: 60	M: 0 F: 60	-	46 (5.0)	45 (6.0)	45 (6.0)	-	N/R
Salo et al. (2010)	60	60	60	-	M: 0 F: 60	M: 0 F: 60	M: 0 F: 60	-	46 (5.0)	45 (6.0)	46 (6.0)	-	No significant difference
Tunwattanapong et al. (2015)	48	48	-	-	M: 5 F: 43	M: 4 F: 44	-	-	36.5 (8.7)	34.2 (9.0)	-	-	No significant difference
Viljanen et al. (2003)	130	135	-	-	M: 0 F: 130	M: 0 F: 135	-	-	44 (7.4)	45 (6.6)	-	-	Control group lower pain score; Intervention group lesser satisfaction with work

*Source*: Authors’ own work

Contr, control; Int, intervention; N/R, not reported; M, male; F, female.

Overall three research groups conducted studies on this topic. A Denmark-based research group published the following articles: Andersen et al. (2008), Andersen et al. (2011), Andersen et al. (2012) and Gram et al. (2014). A Finland-based research group published the following articles: Viljanen et al. (2003), Nikander et al. (2006) and Salo et al. (2010). A Thailand-based research group published the following article: Tunwattanapong et al. (2015). Four studies (Andersen et al. 2008, 2012; Gram et al. 2014; Viljanen et al. 2003) included participants who had experienced neck pain in the last three months preceding the study. Nikander et al. (2006) and Salo et al. (2010) included participants who had constant or frequent neck pain for more than six months. Andersen et al. (2011) only accepted participants with self-reported neck pain greater than 2/10 on VAS for the last three months. Tunwattanapong et al. (2015) included participants with a VAS score greater than 5 for the last three months.

### Study interventions

Similarities in the therapeutic exercise interventions were found across the eight studies as shown in Table 3. Six studies (Andersen et al. 2008, 2011, 2012; Nikander et al. 2006; Salo et al. 2010; Viljanen et al. 2003) included control groups that performed health promoting activities and two studies (Gram et al. 2014; Tunwattanapong et al. 2015) had control groups performing no therapeutic exercise. Viljanen et al. (2003), Andersen et al. (2008), Andersen et al. (2011), Andersen et al. (2012) and Gram et al. (2014) included strengthening exercises in their interventions. Nikander et al. (2006) and Salo et al. (2010) focused their interventions on strengthening and endurance exercises. Tunwattanapong et al. (2015) included stretching as an intervention. The intervention time period ranged from 4 weeks to 12 months. The dosage intervals were similar across the studies as shown in Table 4. Andersen et al. (2008) and Viljanen et al. (2003) also included an all-round exercise intervention group and a relaxation intervention group, respectively. Data from the all-round exercise intervention and the relaxation intervention groups were not included in the review as they do not fall within the scope of this review.

**TABLE 3:** Description of interventions.

Author	Intervention period	Intervention group	Exercise			Control	
			Type	Description	Dosage	Type	Description
Andersen et al. (2008)	12 months	1	Strengthening	Dynamic strengthening exercises from neutral then in 90° shoulder elevation (front raise, lateral raise and shoulder shrugs); static neck exercises in sitting (neck flexion, neck extension and lateral flexion); high-speed power exercise (ergometer)	20 min 3 times/week (once supervised) Dynamic strengthening: 2–3 sets of 10–15 reps Static neck exercise: repetitions of 5s duration at 70% – 80% of MVC Dynamic power exercise: 15s all-out fast speed	Health promotion activities	Improve workplace ergonomics, stress management and organisation of work in a group
Andersen et al. (2011)	10 weeks	1	Strengthening	12 min group: performed progressive resistance exercises for neck/shoulder muscles (lateral raise)	5 times/week, total 60 min/week 5–6 sets of 8–12 reps	Health promotion activities	Weekly e-mailed information on general health with additional relevant information
		2	Strengthening	2 min group: progressive resistance exercises for neck/shoulder muscles (lateral raise)	5 times/week, total 10 min/week 1 set to failure		
Andersen et al. (2012)	20 weeks	1	Strengthening	Dynamic strength exercises (front raise, lateral raise, reverse flies, shrugs and wrist extension with dumbbells)	1 h once/week	No exercise	Maintain level of physical activity and avoid any regular exercise
		2	Strengthening	Dynamic strength exercises (front raise, lateral raise, reverse flies, shrugs and wrist extension with dumbbells)	20 min 3 times/week		
		3	Strengthening	Dynamic strength exercises (front raise, lateral raise, reverse flies, shrugs and wrist extension with dumbbells)	7 min 9 times/week		
Gram et al. (2014)	20 weeks	1	Strengthening	Minimal supervision of 40–60 min Warm up: 10 reps of each exercise with 50% of 1 RM. Specific strength exercise for the neck, shoulder and wrist muscles (front raise, lateral raise, reverse flies, and shrugs with dumbbells)	20 min 3 times/week 20 RM at the beginning of the intervention period to 8 RM during the later phase	No exercise	Maintain level of physical activity and avoid any regular exercise
		2	Strengthening	With supervision of 10 h Warm up: 10 reps of each exercise with 50% of 1 RM. Specific strength exercise for the neck, shoulder and wrist muscles (front raise, lateral raise, reverse flies, and shrugs with dumbbells)	20 min 3 times/week 20 RM at the beginning of the intervention period to 8 RM during the later phase		
Nikander et al. (2006)	12 months	1	Strengthening	Strengthening exercise for neck flexor muscles in sitting (front raise, lateral raises, horizontal flexion, extension, shoulder extension with rubber band); dynamic exercises (shrugs, presses, curls, bent-over rows, flies and pullovers with dumbbells); trunk and legs strengthening using body weight and multimodal rehabilitation; stretching neck, shoulder and upper limb muscles; advised aerobic exercises	Neck exercise: 1 set of 15 reps Dynamic exercises: 1 set of 20 reps with 4 kg–13 kg Trunk and legs: 3 sets of 20 reps Stretching: 3 sets of 9 stretches Aerobic exercises: 30 min 3 times/week	Health promotion activities	Aerobic exercises 3 times/week for 30 min; written information on 9 stretching exercises, 20 min 3 times/week
		2	Endurance	Lift head up from a supine position to exercise neck flexor muscles; dynamic exercises (shrugs, presses, curls, bent-over rows, flies, pullovers with dumbbells); trunk and legs strengthening using body weight and multimodal rehabilitation; stretching neck, shoulder and upper limb muscles; advised aerobic exercises	Neck exercise: 3 sets of 20 reps Dynamic exercises: 3 sets of 20 reps with 2 kg Trunk and legs: 3 sets of 20 reps Stretching: 3 sets of 9 stretches Aerobic exercises: 30 min 3 times/week		
Salo et al. (2010)	12 months	1	Endurance	Lift head up from a supine position to exercise the neck flexor muscles and strengthening exercise for neck muscles (shrugs, presses, curls, bent-over rows, flies, pullovers with dumbbells); encouraged to do aerobic exercise	Neck exercises: 3 sets of 20 reps Dynamic exercises: 3 sets 20 reps with 2 kg Aerobic exercises: 30 min 3 times/week	Health promotion activities	Written information and guidance session on stretching exercises; encouraged to do aerobic exercise 30 min 3 times/week
		2	Strengthening	Strengthening exercise for neck muscles in sitting (shrugs, presses, curls, bent-over rows, flies, pullovers with dumbbells); dynamic strengthening (squats, sit-ups, back extensions). Stretching neck, shoulders and upper limbs; encouraged to do aerobic exercise	Neck exercises: 15 reps, reaching resistance level of 80% of the patient’s maximum isometric strength as recorded at baseline Dynamic exercises: 1 set of 20 reps at maximal weight ability Aerobic exercises: 30 min 3 times/week		
Tunwattanapong et al. (2015)	4 weeks	1	Stretching	Stretching exercises of the neck and shoulder (shoulder rolling, trunk stretching, back extension exercises); handout brochure on proper position and daily ergonomics at work	5 times/week 20–30 reps per session twice a day	No exercise	Brochure indicating proper position and ergonomics; maintain level of physical activity
Viljanen et al. (2003)	12 months	1	Strengthening	Dynamic strengthening exercises for shoulder girdle muscles (front and lateral raises with dumbbell) and static exercises for cervical spinal muscles (shoulder shrugs)	3 times/week, 30 min each 2–3 sets of 10–15 reps with 1 kg–3 kg	Health promotion activities	Improve workplace ergonomics, stress management and organisation of work

*Source*: Authors’ own work

Min, minutes; Reps, repetitions; RM, repetition maximum; MVC, maximum voluntary contraction; Kg, kilogram.

**TABLE 4:** Description of outcome measures.

Authors	Outcome measures for pain	Outcome measures for quality of life	Measurement intervals
Andersen et al. (2008)	Intensity scale (0–9)	-	Baseline, 6 months, 12 months
Andersen et al. (2011)	VAS (0–10) Total tenderness score (0–32)	-	Baseline, 10 weeks
Andersen et al. (2012)	Intensity scale (0–9)	DASH (0–100)	Baseline, 20 weeks
Gram et al. (2014)	Intensity scale (0–9)		Baseline, 20 weeks
Nikander et al. (2006)	VAS scale (0–100 mm )	Modified neck and shoulder pain disability index (0–100)	Baseline, 12 months
Salo et al. (2010)		15D (single 0–1 score)	Baseline, 12 months
Tunwattanapong et al. (2015)	VAS scale (0–100 mm) NPQ (0–100)	SF-36	Baseline, 4 weeks
Viljanen et al. (2003)	Intensity scale (0–10)	NDI (0–80)	Baseline, 6 months, 12 months
		Subjective normal life limitation (0–10)	
		Subjective workability (0–10)	
		Work limited by neck pain (0–100)	

*Source*: Authors’ own work

15D, 15-dimension score; DASH, disability of arms, shoulders and hands index; NDI, neck disability index; NPQ, Northwick Park Neck Pain Questionnaire; SF-36, short Form 36 Questionnaire; VAS, visual analogue scale.

### Study outcome measures

The outcome measures of interest were pain and QoL. All studies excluding Salo et al. (2010) measured pain, using either an intensity scale (Andersen et al. 2008, 2012; Gram et al. 2014) or the VAS (Andersen et al. 2011; Tunwattanapong et al. 2015). Quality of life was assessed in five studies (Andersen et al. 2012; Nikander et al. 2006; Salo et al. 2010; Tunwattanapong et al. 2015; Viljanen et al. 2003. Outcome measures used included DASH (Andersen et al. 2012), Modified Neck and Disability Index (Nikander et al. 2006), 15-Dimension score (15D) (Salo et al. 2010), SF36 (Tunwattanapong et al. 2015) and NDI (Viljanen et al. 2003) (Table 4).

### The effect of therapeutic exercise on pain

#### Strengthening exercise

The baseline and follow-up values for pain, as reported in the studies incorporating strengthening exercise, are shown in Table 5. Five studies (Andersen et al. 2008, 2011, 2012; Gram et al. 2014; Nikander et al. 2006) reported a statistical significant difference post-intervention between the intervention and control groups for neck pain intensity, with *p*-values ranging from 0.05 to 0.0001.

**TABLE 5:** The baseline and follow-up values for pain scores of studies incorporating strengthening exercise.

Author	Group	Baseline Mean(SD)	10 Weeks Mean(SD)	20 Weeks Mean(SD)	6 Months Mean(SD)	12 Months Mean(SD)
Andersen et al. (2008)	Int 1	5.0 (0.2)	-	-	3.4 (0.2)	N/R
	Contr	N/R			N/R	N/R
Andersen et al. (2011)	Int 1	3.9 (2.7)	N/R	-	-	-
	Int 2	3.5 (1.7)	N/R			
	Contr	3.5 (1.7)	N/R			
Andersen et al. (2012)	Int 1	3.32 (2.25)	-	N/R	-	-
	Int 2	3.13 (2.41)		N/R		
	Int 3	3.05 (2.30)		N/R		
	Contr	3.24 (2.26)		N/R		
Gram et al. (2014)	Int 1	2.4 (2.4)	-	1.8 (N/R)	-	-
	Int 2	2.6 (2.5)		1.9 (N/R)		
	Contr	2.5 (2.5)		2.3 (N/R)		
Nikander et al. (2006)	Int 1	5.7 (2.0)	-	-	-	1.8 (2.2)
	Contr	5.8 (2.0)				4.2 (2.3)
Viljanen et al. (2003)	Int 1	4.8 (2.3)	-	-	2.9 (2.8)	3.1 (2.5)
	Contr	4.1 (2.2)			2.9 (2.8)	3.2 (2.5)

*Source*: Authors’ own work

Contr, control; Int, intervention; N/R, not reported; SD, standard deviation.

In terms of neck pain reduction in the study by Andersen et al. (2012), there was no statistical significant difference between any of the three separate intervention groups that varied in training session duration, when individually compared to the control group. However, when grouped together, the intervention group had a significant reduction in neck pain compared to the control group (*p* < 0.05). Furthermore, combining Intervention 1 and Intervention 2 showed a significant difference compared to the control group (*p* < 0.005). In Gram et al.’s (2014) study, Intervention 1 (minimal supervision) had a significant decrease in pain scores compared to the control group (*p* = 0.02). However, the decrease in pain scores for Intervention 2 (with supervision) was not statistically significant (*p* = 0.07). The study by Viljanen et al. (2003) reported no significant difference in neck pain intensity post-intervention between the intervention and control groups.

**FIGURE 2:**
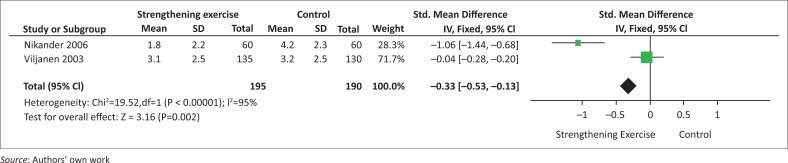
Forest plot of the effect of strengthening exercise versus no therapeutic exercise (control) in improving pain measured at 12 months.

The forest plot in Figure 2 shows the effect of strengthening exercise versus no therapeutic exercise in improving pain measured at 12 months. The comparable data (mean and standard deviation [SD]) for the outcome pain were combined from two studies (Nikander et al. 2006; Viljanen et al. 2003) and the meta-analysis revealed that there is a clinically significant difference between the intervention group performing strengthening exercise and the control group (*p* = 0.002). Heterogeneity in the summary effect of the combined studies was significantly high (*p* < 0.00001). This could be because of the large discrepancy in the study sample sizes, shifting the weighting of the overall effect. The overall effect shows that strengthening exercise can have a significant effect on pain reduction for up to 12 months after the intervention is completed.

#### Endurance exercise

The study by Nikander et al. (2006) was the only study reporting the effect of endurance exercise (Intervention 2 in Table 3) on the neck pain intensity of office workers with non-specific neck pain. The pain scores reported in mean (SD) improved from baseline 57 (21) to post-intervention 23 (22), whilst the control improved from 58 (20) to 42 (23), respectively. Nikander et al. (2006) reported a statistical significant difference between the intervention and the control groups (*p* < 0.001).

#### Stretching exercise

The study by Tunwattanapong et al. (2015) was the only study reporting the effect of stretching exercise on neck pain intensity among office workers with non-specific neck pain. The authors reported a significant improvement in the VAS pain scores from baseline 6.6 (1.2) to post-intervention 4.8 (1.8) within the intervention group compared with the control group (6.2 [1.0] to 5.6 [1.8], post-intervention [*p* = 0.001]) (Tunwattanapong et al. 2015). The Northwick Park Neck Pain Questionnaire (NPQ) score for the intervention group improved from baseline 28.2 (12.0) to 22.2 (11.3) post-intervention and the control group improved from baseline 28.9 (12.5) to 26.7 (14.5), but this was not significant (*p* = 0.055) (Tunwattanapong et al. 2015).

### The effect of therapeutic exercise on quality of life

#### Strengthening exercise

**FIGURE 3:**
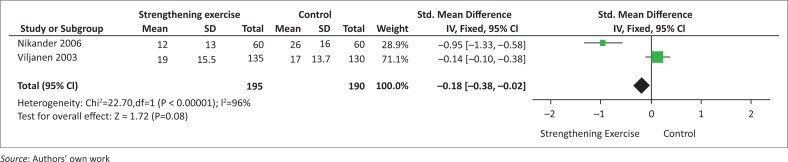
Forest plot of the effect of strengthening exercise versus no therapeutic exercise (control) in improving quality of life measured at 12 months.

The baseline and follow-up values for QoL, as reported in the studies incorporating strengthening exercise, are shown in Table 6. Two studies showed a statistical significant difference in the post-intervention QoL scores between the intervention and control groups (Andersen et al. 2012; Salo et al. 2010). Salo et al. (2010) reported that intervention 2 was significantly different in the QoL scores than the control group (*p* = 0.012). Andersen et al. (2012) reported *p*-values of *p* < 0.05 and *p* < 0.01 for Intervention 1 and Intervention 2, respectively. There was no statistical significant difference between Intervention 3 and the control group in the study. Nikander et al. (2006) and Viljanen et al. (2003) reported no statistical significant difference between the intervention and control groups (no *p*-values reported). However, Nikander et al. (2006) stated that there was a larger reduction in health behaviour modification obtained by the intervention group and not the control group (Table 6). The forest plot in Figure 3 shows the effect of strengthening exercise versus no therapeutic exercise in improving QoL measured at 12 months. The comparable data (means and SD) for the outcome QoL were combined from two studies (Nikander et al. 2006; Viljanen et al. 2003) and the meta-analysis revealed that there is no clinically significant difference between the strengthening intervention group and the control group (*p* = 0.08). Heterogeneity in the summary effect of the combined studies was significantly high (*p* < 0.00001). This could be because of the large discrepancy in the study sample sizes, shifting the weighting of the overall effect. Furthermore, participants reported neck pain for the last three months at baseline in the study by Viljanen et al. (2003), compared to the reported neck pain for the last six months in the study by Nikander et al. (2006).

**TABLE 6:** The baseline and follow-up values for quality of life scores of studies incorporating strengthening or endurance exercise as an intervention compared to their respective controls.

Author	Group	Baseline Mean (SD)	20 Weeks Mean (SD)	12 Months Mean (SD)
Andersen et al. (2012) DASH (0–100)	Int 1	12 (16)	N/R	-
	Int 2	13 (18)	N/R	
	Int 3	10 (16)	N/R	
	Contr	11 (14)	N/R	
Nikander et al. (2006) Modified neck and shoulder pain disability index (0–100)	Int 1	35 (13)	-	12 (13)
	Contr	38 (15)		26 (16)
Salo et al. (2010) 15D Questionnaire (single 0–1 score)	Int 2	0.9034 (0.05854)	-	0.9271 (0.06338)
	Contr	0.9124 (0.04997)		0.9101 (0.05341)
Viljanen et al. (2003) Neck disability index (0–80)	Int 1	29 (15.4)	-	19 (15.5)
	Contr	26 (13.8)		17 (13.7 )
Normal life limited by pain (0–10)	Int 1	2.6 (2.3)		1.5 (2.0)
	Contr	2.3 (2.0)		1.3 (1.8)
Work limited by neck pain (0–10)	Int 1	3.3 (2.3)		2.0 (2.3)
	Contr	2.8 (2.1)		1.5 (1.9)
Subjective workability (0–10)	Int 1	7.7 (1.1)		7.9 (1.2)
	Contr	7.8 (1.2)		8.0 (1.4)
Nikander et al. (2006)	Int 2	38 (14)	-	16 (16)
	Contr	38 (15)		26 (16)
Salo et al. (2010)	Int 1	0.8962 (0.06298)	-	0.9209 (0.05704)
	Contr	0.9124 (0.04997)		0.9101 (0.05341)

*Source*: Authors’ own work

Contr, control; Int, intervention; N/R, not reported; SD, standard deviation.

#### Endurance exercise

The baseline and follow-up values for QoL, as reported in the two studies (Nikander et al. 2006; Salo et al. 2010) incorporating endurance exercise, are shown in Table 6. Both studies reported a statistically significant difference in QoL scores between the intervention and control groups after 12 months. Salo et al. (2010) reported a statistically significant difference of *p* = 0.0019; however, Nikander et al. (2006) did not report a *p*-value.

#### Stretching exercise

Tunwattanapong et al. (2015) found that the SF-36 physical score improved for the intervention group from baseline 53.3 (19.5) to post-intervention 64.3 (18.9), whereas the control group deteriorated from 61.7 (18.5) to 56.8 (19.8). The mental dimension changed from 61.6 (19.1) to 68.9 (19.5) for the intervention group and from 66.6 (18.0) to 67.9 (18.2) for the control group over the entire duration of the study. This study showed statistical differences in QoL scores post-intervention between the intervention and control groups for the SF-36 physical score (*p* < 0.001). The SF-36 mental score was not statistically significant (*p* = 0.127). Furthermore, significantly higher scores were found among those who exercised ≥ 3 times per week (*p* = 0.005) than those exercising less frequently within the intervention group (*p* = 0.018).

## Discussion

This review sought to determine the effectiveness of therapeutic exercise on pain and QoL in office workers with non-specific neck pain compared to no therapeutic exercise. The findings of the review suggest that there is good quality evidence to support strengthening exercise more over endurance and stretching exercises to reduce neck pain intensity and improve QoL in office workers with non-specific neck pain.

Regular strengthening exercise was considered in six studies, where five of them revealed that this type of exercise significantly improves pain intensity in the intervention groups compared to groups receiving no exercise or only health promotional activities. The meta-analysis revealed a clinical significance favouring strengthening exercise in pain reduction; however, the heterogeneity in the summary effect of the combined studies was significantly high because of the large discrepancy in the study sample size, shifting the weighting of the overall effect of the forest plots compiled. Andersen et al. (2012) found no statistically significant difference between the individual intervention groups when compared to the control group; however, when combining the intervention groups, a statistical significance was found. All three intervention groups trained for a total of 1 h per week, but the frequency of training sessions differed between the intervention groups (see Table 4). Therefore, it is possible that the total time spent exercising per week is more important than the frequency of training sessions (Candow & Burke 2007). Andersen et al. (2011) confirmed this as their Intervention 1 had better pain relief by training for a total of 1 h a week, whilst Intervention 2 was less effective in reducing pain scores when training for 10 min a week. Andersen et al. (2012) only did shoulder exercises, whereas Andersen et al. (2008) and Andersen et al. (2011) included specific neck exercises over and above shoulder exercises. A study conducted by Borisut et al. (2013) on exercise for neck pain found that performing exercises for the specific symptomatic region improves pain and disability within that region more than that of general or non-area specific exercises. This could possibly explain why the results reported by Andersen et al. (2012) were not as beneficial to improving neck pain as those reported by Andersen et al. (2008) and Andersen et al. (2011). In the study by Gram et al. (2014), both intervention groups did the same exercises, but only Intervention 1 (minimal supervision) had a significant decrease in pain scores compared to the control group. Exercising with a home programme allows for more flexibility in the training routine (Gram et al. 2014). This result implies that individuals can be sent home to continue with their newly learnt exercises and not lose out on the treatment effect even if not constantly supervised (Savage et al. 2001).

Furthermore, Swenson (2003) confirmed that patients receiving a well-explained and demonstrated home exercise training programme benefitted significantly more than patients receiving general exercise recommendations. There was no statistically significant difference between the intervention and control groups reported by Viljanen et al. (2003). At the commencement of the study by Viljanen et al., the control group had significantly lower pain intensity scores than the intervention group, making the groups incomparable. The findings of this study substantiate the findings of another systematic review on mechanical neck pain disorders, which indicated that exercise is beneficial in the management of neck pain, particularly strengthening exercise focusing on the neck, shoulder and shoulder blade regions (Gross et al. 2015).

Only one study reported on the effect of endurance training on neck pain intensity and found a statistical significant difference favouring the intervention group (Nikander et al. 2006). Tunwattanapong et al. (2015) used stretching as an intervention to manage non-specific neck pain. However, both the VAS and NPQ showed improvement in pain scores, but only the VAS indicated a significant difference (Tunwattanapong et al. 2015). This could be because of the differences in the pain outcome measures used as the NPQ reports on how pain affects nine different aspects of daily living (Misailidou et al. 2010) whilst the VAS only addresses pain intensity.

Five studies considered the effect of regular strengthening exercise on QoL, with four of them indicating the positive results of this intervention. In the study which found no benefit of strengthening exercise on QoL, Viljanen et al. (2003) reported lower work satisfaction at baseline in the intervention group compared to the control group, which influences the external validity of the study. The intervention group had a relatively greater improvement in QoL compared to the control group possibly because of the difference in baseline measurement favouring the control group.

Two studies (Nikander et al. 2006; Salo et al. 2010) considered the effect of endurance exercise on an individual’s QoL and both found a positive effect on QoL compared to no therapeutic exercise. Stretching exercise was found to be significantly beneficial in the physical domain only when compared to the control group in improving QoL (Tunwattanapong et al. 2015). Salo et al. (2010) concluded that the strengthening exercise group showed a greater improvement in QoL than the endurance exercise group. This evidence aligns with the latest systematic review on mechanical neck disorders which suggests the use of strengthening exercise combined with endurance or stretching exercise to reduce neck pain in office workers (Gross et al. 2015). However, minimal effect on neck pain and function was found when only stretching or endurance-type exercises were used for the neck, shoulder and shoulder blade regions (Gross et al. 2015).

The articles in this review have moderate to excellent methodological quality scores according to the PEDro score, providing trustworthy evidence (Hariohm, Prakash & Saravankumar 2015). However, none of the studies implemented blinding of the subjects or the therapists. The observed effects could be because of the placebo effect or because of the therapists’ level of enthusiasm towards the treatment or control conditions. There is potential systematic bias as four of the eight studies did not conceal allocation of participants to the various groups (Andersen et al. 2008, 2012; Gram et al. 2014; Nikander et al. 2006). As mentioned previously, there is a higher prevalence of female office workers with non-specific neck pain, and this is reflected in three of the eight studies which included only female participants (Nikander et al. 2006; Salo et al. 2010; Viljanen et al. 2003). Three studies had a much higher female to male ratio within their sample groups (Andersen et al. 2008, 2011; Tunwattanapong et al. 2015).

The majority of the outcome measurement tools used in the included studies are reliable and valid. The VAS is more sensitive than the NPRS (Misailidou et al. 2010). The Neck Pain Intensity and Total Tenderness Scores are classified as numerical rating scales (Misailidou et al. 2010). The VAS was only used in three of the studies (Andersen et al. 2011; Nikander et al. 2006; Tunwattanapong et al. 2015). This could influence the sensitivity of the results of those studies that did not use the VAS. There is good construct validity for the Modified Neck and Shoulder Pain Disability Index, NDI, NPQ and Vernon NDI (Misailidou et al. 2010). The 15D scores, SF-36 and DASH are highly reliable, sensitive, responsive to change and generalisable in Western-type societies (Beaton et al. 2001, Sintonen 2001; Zhang et al. 2012). Viljanen et al. (2003) used outcome measures to describe QoL which have not been validated previously and this could potentially be the reason for their results not indicating a favourable outcome.

In keeping with the findings of this review, the following recommendations can be made for the clinical setting. There is strong evidence suggesting that strengthening exercise for a total of 1 h per week reduces the intensity of neck pain and improves QoL in office workers with non-specific neck pain. There is not enough substantial evidence that supports endurance and stretching exercise to reduce pain and improve QoL; however, combining these interventions with the strengthening treatment regime may improve the patient’s overall outcome.

## Limitation and recommendations

Studies published in a language other than English were excluded and this could have potentially excluded eligible articles from the review. Further studies need to be conducted around the world, particularly in developing countries, to improve the global application of this evidence. Further studies should be conducted on the effectiveness of endurance exercise on pain to draw conclusive evidence. There was a variety of outcome measurement tools used, which made it challenging to combine and compare study results. Therefore, similar standardised outcome measurement tools should be used to allow for comparisons between studies and meta-analysis on comparable data. There were limited randomised control trials investigating the effectiveness of endurance and stretching exercise as interventions for treating non-specific neck pain in office workers. Future studies of high quality on all interventions have to be conducted. There is currently a lack of studies that measure QoL and function of office workers with non-specific neck pain. It is recommended for researchers to incorporate QoL and functional measures as determinants in the patient’s recovery process.

## Conclusion

In summary, there is level II evidence suggesting that strengthening exercise therapy can improve pain and QoL in office workers with non-specific neck pain. However, there is not enough substantial evidence to support the effects of endurance exercise or stretching alone to improve pain and QoL. Clinicians are encouraged to include 1 h of weekly strengthening exercise for the shoulder and neck regions to address pain and QoL in office workers with non-specific neck pain. Further research needs to be implemented to address effects of endurance and stretching exercise on QoL and pain in office workers with non-specific neck pain.
